# Effects of Hydroxyapatite-Containing Toothpastes on Some Caries-Related Variables: A Randomised Clinical Trial

**DOI:** 10.1016/j.identj.2024.01.028

**Published:** 2024-03-06

**Authors:** Guglielmo Campus, Fabio Cocco, Richard Johannes Wierichs, Thomas Gerhard Wolf, Claudia Salerno, Antonella Arghittu, Marco Dettori, Maria Grazia Cagetti

**Affiliations:** a Department of Restorative, Preventive and Pediatric Dentistry, University of Bern, Bern, Switzerland; b Department of Surgery, Microsurgery and Medicine Sciences, School of Dentistry, University of Sassari, Sassari, Italy; c Department of Cariology, Saveetha Dental College and Hospitals, SIMATS, Chennai, India; d Department of Periodontology and Operative Dentistry, University Medical Center of the Johannes Gutenberg-University Mainz, Mainz, Germany; e Department of Biomedical, Surgical and Dental Sciences, University of Milan, Milan, Italy; f Graduate School for Health Sciences, University of Bern, Switzerland

**Keywords:** Caries, Children, Fluoride, Nano-hydroxyapatite, Randomised clinical trial, Toothpaste

## Abstract

**Objectives:**

This randomised clinical trial was designed and carried out with the aim to evaluate the capacity of fluoride-substituted hydroxyapatite (HAF) toothpaste to modulate oral microflora composition and biofilm acidogenicity in schoolchildren.

**Methods:**

In all, 610 children (4 to 5 and 6 to 7 years) were enrolled. Four toothpastes were randomly administered during 24 months: 2 contained fluoride-substituted hydroxyapatite (HAF_1000_ and HAF_1450_; 1000 and 1450 ppmF) and magnesium-, strontium-, and carbonate-substituted hydroxyapatite in a chitosan matrix, and 2 were monofluorophosphate fluoridated toothpastes (F_1000_ and F_1450_; 1000 and 1450 ppmF). Caries lesions were assessed by International Caries Detection and Assessment System scores, supragingival plaque was sampled from the approximal sites between primary molars using sterile Gracey curettes for microbiological analysis, and plaque pH curves after sucrose challenge were assessed at baseline and reevaluated after 1 year and after 2 years. The minimum and maximum pH decrease was calculated for caries-free patients and participants with a caries lesion(s) at baseline and at the end of the experimental period (24 months). Differences amongst measurements were analysed with 1-way analysis of variance.

**Results:**

During the trial, the minimum pH value increased statistically significantly in all groups; in HAF_1000_ and HAF_1450_, the increase was greatest. At the end of trial, in the 2 HAF groups all primary cariogenic bacteria were statistically significantly lower with respect to F groups (*P* = .03 for *Streptococcus mutans* and *sobrinus*, for *Lactobacillus casei*, and for *Lactobacillus fermentum*).

**Conclusions:**

The trial provides robust but still inconclusive evidence on the efficacy of HAF toothpastes compared to traditional fluoridated toothpastes to reduce caries risk factors and to prevent caries lesions.

## Introduction

Nano-hydroxyapatite (n-HAp) toothpaste is a type of toothpaste that contains nanoparticles of hydroxyapatite, a naturally occurring mineral form of calcium apatite, which is the main component of tooth enamel. Hydroxyapatite has been widely studied and recognised for its ability to remineralise tooth enamel and improve dental health.^1^

The composition of the oral microbiota influences the oral ecosystem's balance and plays a pivotal role in maintaining oral health. Monitoring the levels of cariogenic bacteria in plaque or saliva can help assess the risk of future caries.^2^^,^^3^ The acid formation by cariogenic bacteria is a critical event in the development of carious lesions, and frequent and prolonged decreases in plaque pH have been found to be associated with caries activity at both the individual^4^^,^^5^ and surface^6^ level.

Monitoring plaque-pH levels helps in understanding the acidity of the oral environment and its potential to cause caries lesions. Regular assessment of plaque pH can provide insights into the dynamics of acid production and the effectiveness of preventive measures, such as oral hygiene practices and dietary modifications. A physiologic salivary flow and a proper buffering capacity can neutralise acid attacks, remineralise enamel, and wash away food particles, reducing the risk of dental caries. Hence, the evaluation of these parameters, such as plaque pH, salivary flow rate, and microbial composition, can provide valuable information about individuals’ susceptibility to caries development.^7^, ^8^, ^9^ In combination with other clinical assessments, these parameters enable dental professionals to assess patients' caries risk and plan tailored preventive strategies accordingly. In addition, the measurement of these factors can aid in early detection and intervention to prevent or minimise the progression of dental caries.

The hypothesis of this study was that administration of HAF toothpaste would be able to modulate oral microflora composition and biofilm acidogenicity in schoolchildren. Furthermore, as a secondary aim, the difference between the biofilm acidogenicity values in relation to the increase in caries was evaluated. The null hypothesis was that HAF toothpaste would not modify oral health–related variables compared to conventional fluoridated toothpaste.

## Materials and methods

The study was protocolled as a randomised, triple-blind clinical trial in which the patient, operator, and evaluator were masked to group allocation.^10^ An independent monitor kept the code and did not break it until the statistical analysis was finalised. A third researcher, who was not involved in the evaluation process, was responsible for the randomisation process.

The study was carried out at the Dental Clinic of University of Sassari (ethical committee approval No. 217/2017 Sassari) and registered at ClinicalTrials.gov (NCT04906291). Regarding the study population, the Italian National Institute for Statistics website (https://www.istat.it) provided the number of children aged 4 to 7 years living in the area as 13,239. The natural fluoride concentration in tap water of the district is 0.04 mg/L (http://www.abbanoa.it/distretto-6).

Children whose birthday fell between September 2012 and June 2013 who were attending kindergarten and children whose birthday fell between September 2010 and June 2011 who were attending primary school were eligible to participate. More information on sample characteristics and inclusion and exclusion criteria can be found in a previous publication.^10^

Power analysis was performed using G*Power 3.1.3 for Apple using a nonparametric Mann–Whitney *U* test, with an effect size of 0.25 and an error probability of 0.05. The number of participants in each age group was set at 256 (504 participants in total), with an actual power of 0.95. The sample size was increased by 15% to safeguard the estimates against the possible number of nonresponders.

A letter explaining the purpose of the study and the informed consent was distributed to the parents/caregivers of children of the 2 age groups considered. A total of 610 participants fulfilling the criteria of inclusion agreed to participate.

Each class was classified as a cluster to facilitate randomisation and the running of the trial. Randomisation was carried out using systematic cluster sampling; each class was identified and listed as a cluster and 4 groups (2 for each age group) were created. The first cluster was selected randomly, whilst the others were selected at systematic intervals of 3 classes. The number of participants was approximately the same in each class (range, 14–16 children).

This study of human participants was in accordance with local legislation and institutional requirements. All performed procedures were in accordance with the ethical standards of the 1964 Helsinki Declaration and its later amendments. The dataset generated and/or analysed during the current study are not publicly available due to data holder restrictions. The data are available on request.

### Study design

**Fig. 1:**
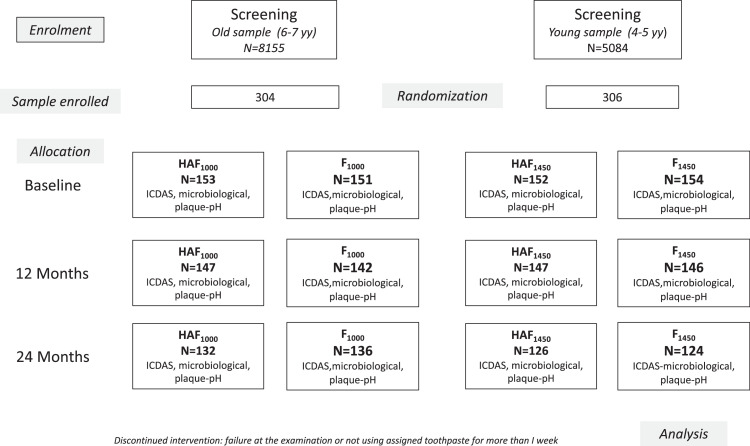
Consort flowchart of the study.

The clinical trial was supposed to be carried out from February 2018 to June 2020. During the second year of the trial, the COVID-19 pandemic reached Italy and on 9 March 2020, the government of Italy imposed a national lockdown or quarantine, restricting the movement of the population except for necessity, work, and health circumstances, in response to the growing pandemic of COVID-19 in the country. At that time, more than 10% of the children in the enrolled sample had not completed the study. As a consequence, some of the follow-up examinations were performed in September and October 2020. The design of the study (CONSORT flowchart) is displayed in Figure 1. The CONSORT checklist is presented in the supplementary file.

The clinical examination was repeated after 12 months and at the end of the experimental period (24 months or longer due to the COVID-19 pandemic). As described in the previous publication,^10^ two age groups were created: a younger group (4–5 years at baseline) and an older age group (6–7 years at baseline).

In addition, a standardised questionnaire was administered to parents/caregivers at baseline to obtain information on caries risk factors,^11^^,^^12^ such as behavioural habits (toothbrushing frequency, use of fluoride); dietary habits (use of pacifier at night, number of meals, cariogenic content of food); socioeconomic status of children/family/caregivers, categorised according to the SocFam scale^12^ as medium-low, medium, and medium-high level; and lifestyle behaviours (frequency of dental checkups).

### Clinical examination

Participants were examined at school using a mouth mirror and a World Health Organization–Community Periodontal Index probe under optimal lighting (each examiner wearing an LED headlight); the teeth were cleaned and dried with a piece of gauze prior to the examination. Caries lesions and the number of filled and missing teeth for caries were recorded at tooth level using the International Caries Detection and Assessment System (ICDAS) as initial or moderate or extensive lesions.^13^, ^14^, ^15^ As the clinical examination was conducted in a school setting, the ICDAS code 1 was merged with ICDAS code 2. The calibration of the examiners was previously extensively described^10^ and is briefly summarised here. The examiners were calibrated prior to the start of the trial and before the follow-up examinations.

At baseline, at the 12-month examination, and at the last follow-up examination, good inter- and intra-examiner reliability was recorded amongst the 4 examiners for sound teeth and initial moderate and severe lesions. Oral hygiene habits, dietary behaviours, socioeconomic status, dental checkup frequency, and caries status of the children enrolled in the trial in the 2 groups recorded at baseline are displayed in the Supplementary Materials (Table S1).

### Bacterial plaque samples

The children refrained from eating/drinking 1 hour before bacterial and plaque-pH samples. No tooth-brushing or other tooth-cleaning methods were allowed on the morning of the measurement day. Pooled supragingival plaque was sampled from the approximal sites between teeth 55/54, 64/65, 74/75, and 85/84 using sterile Gracey curettes. If one of the teeth in the approximal site was missing, an adjacent site was used. Each plaque sample was placed in an Eppendorf tube containing 150 μL sterile TE buffer (10 mM Tris-HCl, 1 mM EDTA, pH 7.6). Then 100 μL 0.5 M NaOH was added to the plaque pellet and the bacterial suspension was stored at −20 °C until further processing.^17^

Microbiological analysis was performed using the checkerboard DNA-DNA hybridisation method. Whole genomic probes were matched from 9 bacterial strains grouped in primary cariogenic bacteria (*Streptococcus mutans, Streptococcus sobrinus, Lactobacillus casei*, and *Lactobacillus fermentum*) and bacteria known not to be primarily associated with caries (*Streptococcus mitis, Streptococcus gordoni, Lactobacillus salivarius, Streptococcus sanguinis*, and *Streptococcus salivarius*). Matching the obtained signals with the ones generated by the pooled standard samples, containing a count of 10^6^ and 10^5^ of each bacterial species, respectively, an evaluation of the bacterial count was performed in the samples.^18^^,^^19^

### Plaque acidogenicity

Immediately after the bacterial plaque samples were assessed, plaque acidogenicity was measured using the pH indicator strips in the interproximal space between the children's first and second maxillary primary molars right and left.^20^, ^21^, ^22^ The strips measure a pH value in the range of 4.0 to 7.0 (Spezialindikator, pH range 4.0–7.0; Merck). Each strip was cut into 4 pieces (approximately 2 mm wide) to get a strip that more easily could be inserted into the interproximal space. The strip was held in situ for 10 seconds, after which it was removed and its colour compared to the colour index scheme supplied by the manufacturer. The pH was determined to one decimal of the value. For each site, 3 measurements were carried out. Measurements were performed before (0 minutes) and at 2, 5, 10, 15, and 30 minutes after a mouth rinse with 10% sucrose for 1 minutes.

### Treatment

A total of 610 participants were screened and enrolled in the study; the toothpastes were supplied by Curasept S.p.A. The toothpastes were 2 HAF toothpastes (1000/1450 ppmF) containing fluoride-substituted hydroxyapatite (HAF_1000_ and HAF_1450_) and magnesium-, strontium-, and carbonate-substituted hydroxyapatite in a chitosan matrix and 2 sodium monofluorophosphate fluoridated toothpastes (F_1000_ and F_1450_). The 1000 ppmF toothpastes were administered to the younger children (a pea-sized amount), whilst the 1450 ppmF toothpastes were administered to the older children (a normal full load).^16^

The children were instructed to brush their teeth with a manual toothbrush for at least 2 minutes after each main meal (3 times a day): once at school under the supervision of a teacher and twice at home. Compliance, observed side effects of the products, and possible use of other fluoridated products (ie, salt, gels, mouthwashes) or other brushing/floss procedures (ie, rotary toothbrush and dental floss), which were not allowed throughout the study, were monitored by means of a questionnaire administered to the parents of the participants every month during the study. If a lapse of more than 1 week in the use of the allocated toothpaste was reported or if the use of another fluoridated product was found for a similar period, the child was withdrawn from the study. Adherence and any side effects of the products were assessed by means of a questionnaire administered to the parents of the children every month for the duration of the experiment. The same questionnaire also investigated the possible use of other fluoridated products (eg, salt, gels, mouthwashes), which were not allowed throughout the study. If a lapse of more than 1 week in the use of the assigned toothpaste was reported or if the use of another fluoridated product was found during a similar period, the participant was excluded from the study. To assess the success of the trial, participants were given 2 months' worth of toothpaste at a time and asked to return the empty pack when they received the new pack for the following months. During the exclusion period, one of the authors (CS) contacted all the children and their families via Zoom 3 times a week to check that the toothpastes were being used correctly.

### Statistical analysis

The mean pH of the pH readings, registered in the 2 sites at 5 different time points, was calculated and the mean for the 2 sites at the individual time points was calculated. For each pH curve, minimum pH and maximum pH decrease were recorded. The area under the curve (AUC), as the area between reference pH (6.2 and 5.7) and the pH curve, was computed.^23^

Microbiological analysis was coded on a range of 0 to 5: 0 = no signal; 1 = signal density lower than the low standard (<10^5^ bacteria); 2 = signal density equal to the low standard (=10^5^ bacteria); 3 = signal density higher than the low standard but lower than the high standard (>10^5^ but <10^6^ bacteria); 4 = signal density equal to the high standard (=10^6^ bacteria); and 5 = signal density higher than the high standard (>10^6^ bacteria).

Caries data were grouped as follows: healthy/caries-free participants (ICDAS 0) and participants with caries lesion(s) (ICDAS 1–6). The pH change and minimum and maximum pH decrease were then calculated for children with a new caries lesion within the observation period and for children without a new caries lesion at the end of the experimental period (≥24 months). Differences amongst measurements were analysed with 1-way analysis of variance.

All data were input into a spreadsheet (Microsoft Excel 2021 for Mac, version 16.4.8). Statistical analyses were performed using Stata/SE1 software, version Stata/SE 17.1 for Mac (Intel 64-bit). The efficacy of the treatment was assessed for those who fully followed the protocol (per-protocol participants). For all statistical analyses, the statistical significance was set at *α* = 0.05.

## Results

The plaque pH measurements and minimum pH and maximum pH decrease (Table 1) at baseline were not statistically different amongst the 4 groups (*P* = .14 for minimum pH and *P* = .61 for maximum pH decrease). Within each group during the trial, the minimum pH value increased significantly; in the 2 groups treated with the hydroxyapatite toothpaste, the increase was greatest and was statistically significant. The maximum drop in pH during the trial was statistically significant in participants using the hydroxyapatite toothpaste (HAF_1000_ group and the HAF_1450_ ppmF group) but not for the participants using monofluorophosphate fluoridated toothpastes (*P* = .05 in the F_1000_ group and *P* = .11 in the F_1450_ group).

**Table 1:** Plaque pH measurements (minimum pH and maximum pH decrease) amongst the 4 groups.

	Minimum pH					Maximum pH decrease				
	HAF_1000_	F_1000_	HAF_1450_	F_1450_	*P* value*	HAF_1000_	F_1000_	HAF_1450_	F_1450_	*P* value*
Baseline	5.32 ± 0.41	5.34 ± 0.50	5.35 ± 0.44	5.36 ± 0.37	.14	1.17 ± 0.42	1.20 ± 0.46	1.18 ± 0.56	1.19 ± 0.51	.61
1 year	5.49 ± 0.24	5.44 ± 0.47	5.47 ± 0.48	5.43 ± 0.43	.15	1.03 ± 0.28	1.09 ± 0.74	1.06 ± 0.53	0.98 ± 0.29	.51
2 years	5.72 ± 0.22	5.49 ± 0.32	5.66 ± 0.36	5.51 ± 0.38	.02	1.00 ± 0.34	1.06 ± 0.328	1.05 ± 0.11	1.01 ± 0.34	.28
*P* values	**.01**	**.01**	**.01**	**.01**		**.01**	.05	**.03**	.11	

⁎ One-way analysis of variance PPM of fluoride.

The microbiological concentrations recorded after the checkerboard DNA-DNA hybridisation method at baseline were not statistically significant amongst the 4 groups. In all groups, both primary and nonprimary cariogenic bacteria decreased (nonsignificantly) during the experimental period. At the end of trial, in both HAF groups all primary cariogenic bacteria were statistically significantly lower compared to both F groups at the end of the experimental period (*Streptococcus mutans* and *sobrinus, Lactobacillus casei* and *fermentum)* (Table 2). The highest decrease was observed for *Streptococcus mutans* in the HAF_1000_ group (3.11 ± 1.13 at baseline and 2.00 ± 0.54 at the 2-year follow-up examination). The mean value of the minimum pH after a sucrose challenge was statistically significantly different between the respective HAF and F groups regarding healthy/caries-free participants (ICDAS 0) and also participants with caries lesion(s) (ICDAS 1–6) (1000 ppmF toothpastes and 1450 ppmF toothpastes). The mean values of maximum pH decrease were statistically significant different only in the 1000 ppmF groups in children with caries (Table 3).

**Table 2:** Mean and standard deviation of the scores for the 9 bacterial strains obtained by the checkerboard DNA-DNA hybridisation method amongst the 4 groups.

	Baseline					1 year					2 years				
Strains	HAF_1000_	F_1000_	HAF_1450_	F_1450_	*P* value*	HAF_1000_	F_1000_	HAF_1450_	F_1450_	*P* value*	HAF_1000_	F_1000_	HAF_1450_	F_1450_	*P* value*
Primary cariogenic bacteria															
*S mutans*	3.11 ± 1.13	3.04 ± 1.05	3.19 ± 1.20	3.14 ± 1.11	.08	2.16 ± 0.46	2.24 ± 0.56	2.10 ± 0.77	2.19 ± 0.61	.09	2.00 ± 0.54	2.10 ± 0.40	2.18 ± 0.80	2.21 ± 0.49	**.03**
*S sobrinus*	1.86 ± 0.74	2.00 ± 0.65	1.79 ± 0.70	1.94 ± 0.82	.11	1.76 ± 0.70	1.65 ± 0.60	1.47 ± 0.66	1.50 ± 0.70	**.04**	1.22 ± 0.57	1.41 ± 0.53	1.41 ± 0.27	1.48 ± 0.51	**.03**
*L casei*	2.33 ± 1.81	2.28 ± 0.39	2.20 ± 1.93	2.24 ± 1.05	.09	1.76 ± 0.74	1.54 ± 0.66	1.80 ± 0.65	1.65 ± 1.05	.05	1.50 ± 0.51	1.44 ± 0.74	1.80 ± 0.65	1.65 ± 1.05	**.02**
*L fermentum*	2.42 ± 1.04	2.38 ± 1. 90	2.36 ± 0.90	2.40 ± 1.06	.08	2.06 ± 0.56	2.05 ± 1.05	2.11 ± 0.32	2.09 ± 0.36	.10	1.84 ± 0.32	1.86 ± 1.25	2.09 ± 0.29	2.08 ± 0.45	**.04**
Not primary cariogenic bacteria															
*S sanguinis*	2.47 ± 0.86	2.50 ± 0.86	2.52 ± 0.47	2.49 ± 0.83	.12	2.14 ± 0.82	2.05 ± 0.81	2.27 ± 0.71	2.10 ± 0.65	.09	1.91 ± 067	1.95 ± 1.01	2.18 ± 0.92	2.08 ± 0.84	.10
*S salivarius*	2.56 ± 0.50	2.54 ± 1.01	2.44 ± 0.69	2.48 ± 0.73	.08	2.19 ± 0.61	2.12 ± 0.62	2.21 ± 0.56	2.12 ± 0.48	.15	2.09 ± 0.67	1.81 ± 0.98	2.14 ± 0.44	2.08 ± 0.67	.08
*S mitis*	2.22 ± 0.47	2.21 ± 0.41	2.26 ± 1.08	2.26 ± 1.11	.20	1.72 ± 0.73	1.80 ± 0.75	1.68 ± 0.65	1.78 ± 0.72	.11	1.54 ± 0.58	1.76 ± 0.71	1.62 ± 0.29	1.69 ± 1.05	.20
*S gordonii*	2.46 ± 0.38	2.41 ± 0.74	2.44 ± 0.89	2.41 ± 0.56	.14	2.32 ± 0.94	2.34 ± 0.56	2.35 ± 0.49	2.37 ± 0.67	.14	2.05 ± 0.72	2.31 ± 0.91	2.35 ± 0.51	2.40 ± 0.71	.14
*L salivarius*	2.09 ± 0.62	2.11 ± 0.70	2.12 ± 0.62	2.12 ± 0.48	.24	2.05 ± 1.05	2.08 ± 0.56	2.04 ± 1.17	2.09 ± 1.15	.25	2.01 ± 0.72	2.09 ± 0.63	2.10 ± 0.91	2.08 ± 0.95	.24

⁎ One-way analysis of comparison for mean comparisons amongst the groups within one time point.

**Table 3:** Baseline and follow-up values of minimum and maximum pH decrease amongst the 4 groups in caries-free (ICDAS = 0) and affected (ICDAS = 1/6) children.

	Minimum pH				Maximum pH decrease			
	*Baseline*		*2-year follow-up*		*Baseline*		*2-year follow-up*	
	*Caries-free (ICDAS = 0)*	*Caries (ICDAS = 1/6)*	*Caries-free (ICDAS = 0)*	*Caries (ICDAS = 1/6)*	*Caries-free (ICDAS = 0)*	*Caries (ICDAS = 1/6)*	*Caries-free (ICDAS = 0)*	*Caries (ICDAS = 1/6)*
**HAF (1000)°**	5.58 ± 0.43	4.91 ± 0.52	6.03 ± 0.29	5.56 ± 0.40	*0.83* ± 0.50	*1.24* ± 0.40	*0.78* ± 0.38	*1.20* ± 0.36
**F (1000)°**	5.47 ± 0.41	4.85 ± 0.54	5.62 ± 0.44	5.24 ± 0.35	*0.86* ± 0.52	*1.28* ± 0.51	*0.86* ± 0.37	*1.25* ± 0.31
P value*	*.08*	*.10*	***.01***	***.03***	*.20*	*.10*	*.07*	*.08*
**HAF (1450)°**	5.28 ± 0.40	5.45 ± 0.46	5.71 ± 0.37	5.84 ± 0.39	*0.78* ± 0.34	*1.25* ± 0.38	*0.81* ± 0.13	*1.19* ± 0.18
**F (1450)°**	5.31 ± 0.38	5.51 ± 0.46	5.38 ± 0.38	5.51 ± 0.42	*0.81* ± 0.45	*1.27* ± 0.51	*0.83* ± 0.32	*1.27* ± 0.36
P value*	*.15*	*.13*	***.01***	***.01***	*.22*	*.20*	*.21*	***.04***

⁎ One-way analysis of variance PPM of fluoride. ICDAS, International Caries Detection and Assessment System.

**Fig. 2:**
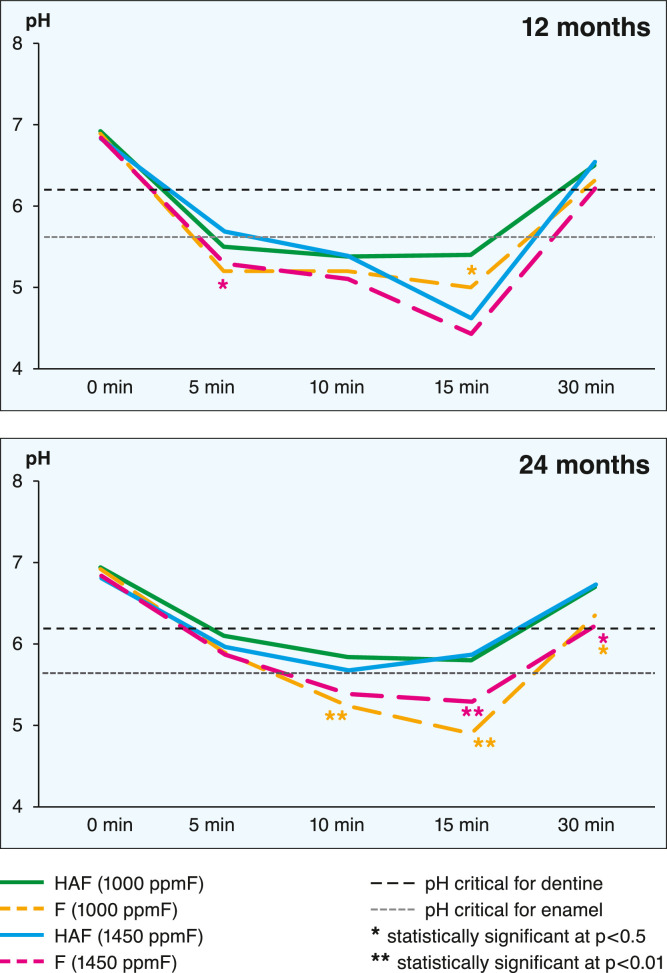
The pH values curves at the 12-month and 24-month examinations in the 4 groups.

The pH value curves at the 12-month and 24-month examinations (Figure 2) showed statistically significant differences between HAF (1000 ppmF) and F (1000 ppmF) at the 12-month examination. At the end of the experimental period, both fluoride concentrations of HAF and F were significantly different (p <0.05).

## Discussion

The present study was designed and carried out to evaluate whether the administration of HAF toothpaste would be able to modulate oral microflora composition and biofilm acidogenicity in schoolchildren. The null hypothesis was that HAF toothpaste would not modify oral health–related variables compared with fluoridated toothpaste. The main results of the study confirm the hypothesis by rejecting the null hypothesis; the administration of HAF toothpaste was able to modify the oral biofilm towards a less cariogenic microflora.

For caries prevention and management of caries, it is mandatory to control its contributing factors. The use of fluoridated toothpastes can increase hard tissue remineralisation, reduce plaque-pH decrease, and decrease microorganisms in the dental plaque.^24^ Three fluoride salts are added in toothpastes—stannous fluoride (SnF_2_), sodium mono-fluorophosphate (Na_2_PO_3_F), and sodium fluoride (NaF)—and all are effective to prevent and manage caries.^25^

Biomimetic HA toothpaste enriched with a chitosan matrix is efficacious in caries prevention and management both in vitro^26^^,^^27^ and in vivo^10^ but there is insufficient evidence on their efficacy in mitigating cariogenic biofilm development. The presence of chitosan in the HAF toothpaste might have influenced the antibacterial activity although the exact mechanism of its antibacterial activity is yet unclear. The most widely accepted hypothesis is that chitosan has the capacity to bind the bacterial cell wall, causing disruption of the cell and thus altering the membrane permeability,^28^ followed by attachment to DNA, causing inhibition of DNA replication and subsequently cell death.^29^ Another possible mechanism is that chitosan acts as a chelating agent that electively binds to trace metal elements, causing toxin production and inhibiting microbial growth.^30^

The antibacterial activity of HAF should also not be underestimated when highlighting the trial results obtained. In vitro, teeth specimens treated with HAF toothpaste showed less early colonisation from cariogenic bacteria with respect to traditional toothpaste,^10^^,^^31^ even if the data in the scientific literature are still inconclusive; this finding is probably linked to the presence of fluoride into the toothpaste formulation.

Regarding the secondary aim—the acidogenicity of the biofilm in the HAF groups was statistically significantly lower compared with F groups—the areas under the curve both for enamel and dentine dissolution were less pronounced in HAF groups at the end of the trial compared with those of F groups.

The use of pH strips to measure the acidogenicity of dental plaque after sucrose challenge and to related to caries status has been investigated^22^; the minimum plaque-pH value is strongly correlated to the number of initial carious lesions.^32^ The pH response of human dental plaque to sugar in vivo has indicated that resting plaque pH, prior to sugar exposure, and minimum pH, reached after sugar exposure, became increasingly lower with increasing caries activity.^21^^,^^33^

This study holds some peculiar characteristics—like the large population enrolled in the trial, the length of the administration, and the quite long follow-up period but, above all, the evaluation for the first time of the effect of an HAF toothpaste on the dental plaque biofilm—reinforcing the hypothesis that HAF toothpastes are able to reduce caries progression, decreasing the number of mutans streptococchi and consequently decreasing aldo the production of acids and the caries increment.

On the other hand, as was previously described,^10^ a limitation of the present trial may be the postponement of the last follow-up due to the COVID-19 pandemic. The suspension of regular school activities may have resulted in less adherence to the protocol and a lack of supervised tooth-brushing, which may have biased the findings. Furthermore, brushing—and therefore the use of the different toothpastes—was carried out twice a day at home, with no possibility for the authors to check for regular adherence to the protocol. It is thus questionable whether the sample selected is representative of the general population of children in this age group.

In Italy, as in several European countries, dental care is not covered by the national health system, and therefore it is of great importance to promote effective low-cost caries prevention strategies. The outcomes of this trial might be generalised, as toothpastes containing fluoride are still the most cost-effective and affordable way to prevent dental caries, especially in countries where other community-level preventive strategies of some effectiveness—such as water fluoridation—are not implemented. Toothpastes containing new agents with remineralising and antibacterial properties, combined with fluoride, could contribute to the control of dental caries, which is still a public health problem in many countries lacking an adequate response from public health authorities. The present randomised clinical trial contributes to the promotion of this type of low-cost intervention.

In conclusion, the present study provides robust but still inconclusive evidence on the efficacy of HAF toothpastes compared with traditional fluoridated toothpastes to reduce caries risk factors and to prevent caries lesions; thus, it is possible to accept the study's hypothesis.

## Conflict of interest

None disclosed.

## Appendix Supplementary materials

Image, application 1

## Footnotes

Supplementary material associated with this article can be found in the online version at doi:10.1016/j.identj.2024.01.028.
